# Open Carpal Tunnel Release With a Z-plasty Rearrangement for Median Nerve Mononeuropathy Secondary to Traumatic Scar Contracture

**DOI:** 10.7759/cureus.39802

**Published:** 2023-05-31

**Authors:** Peter Laub, Jeewon Chon, Sonya Agnew, Kenneth Schiffman, Joseph Ogrodnik

**Affiliations:** 1 Plastic and Reconstructive Surgery, Loyola University Medical Center, Chicago, USA; 2 Orthopaedic Surgery, Loyola University Medical Center, Chicago, USA

**Keywords:** median neuropathy, extraneural, mononeuropathy, hand surgeon, carpal tunnel syndome, open carpal tunnel release, z-plasty

## Abstract

We present the case of a 56-year-old woman who developed carpal tunnel syndrome and palmar scar contracture secondary to a left-hand palmar laceration in a pedestrian versus motor vehicle accident. The patient underwent carpal tunnel release and a Z-plasty rearrangement to restore normal thumb movement. The patient reported significant improvement in thumb mobility, resolution of median neuropathy symptoms, and no pain along the scar at her three-month follow-up. Our case illustrates the effectiveness of a Z-plasty in relieving tension along scars and potential management for traction-type extraneural neuropathy arising from scar contracture.

## Introduction

Carpal tunnel syndrome (CTS) is a compression neuropathy of the median nerve. It is caused by traumatic injury or may be associated with pregnancy, or rheumatological or endocrine disorder [1]. We present the case of a left-hand palmar laceration which resulted in CTS and palmar scar contracture that formed between the base of the thenar eminence through the hypothenar eminence that limited abduction and extension of the thumb. We approached the carpal tunnel through the prior scar and performed a local skin arrangement with a Z-plasty to restore normal thumb movement. 

## Case presentation

**Figure 1:**
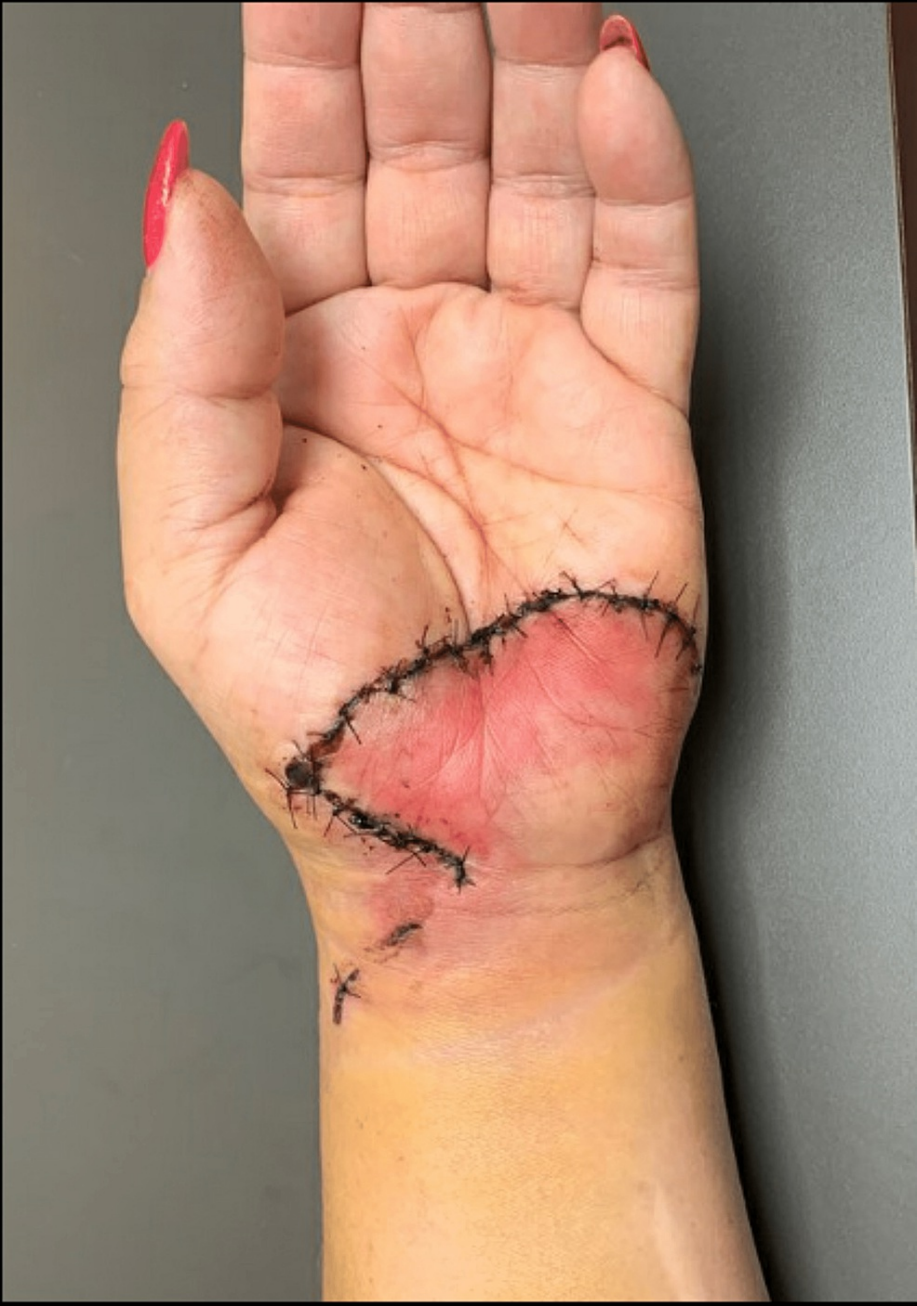
One week after injury Initial presentation to the orthopaedic hand surgeon. The injury was initially sutured by the ED physician.

**Figure 2:**
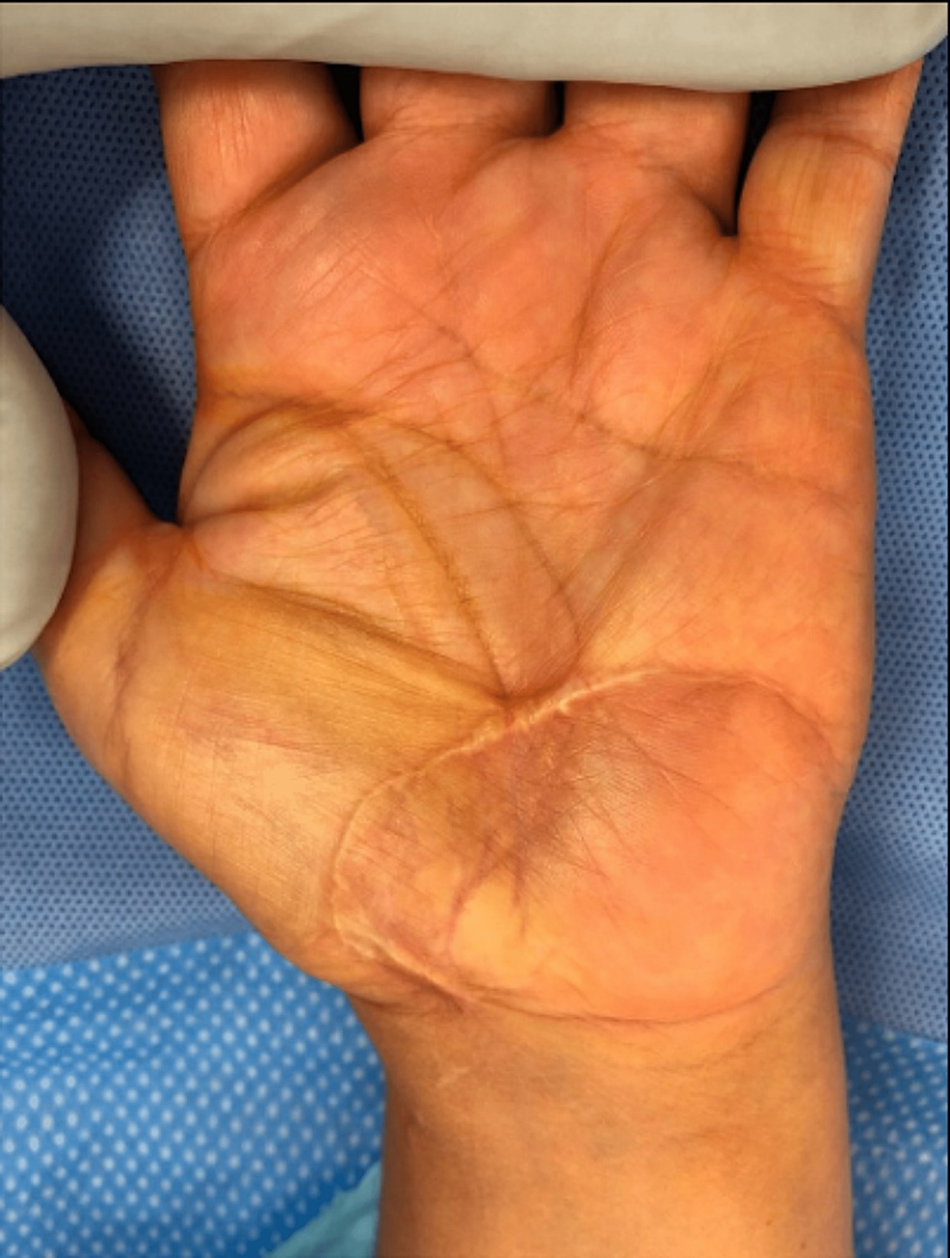
Eight months post injury Scar contracture in the central palm limited thumb abduction and extension.

A 56-year-old left-hand dominant female was evaluated for a left palm hypertrophic scar revision at the same time as carpal tunnel release. The patient sustained a traumatic laceration to the left palm in a pedestrian versus auto accident approximately eight months prior to presentation (Figure 1). The laceration affected the palmar skin and fascia while sparing bony, neurovascular, and tendinous structures. After the initial injury, the patient was prescribed occupational therapy which included range of motion (ROM) and strengthening exercises. Despite several months of hand therapy, the patient developed a painful scar contracture across the base of the thenar eminence through the hypothenar eminence that prevented full abduction and extension of the thumb (Figure 2). 

Four weeks after the injury, the patient complained of intermittent numbness and tingling in the thumb, index, and middle fingers. The patient did not have any CTS symptoms prior to the injury. Initially, the numbness and tingling were thought to be from edema and would likely resolve. The patient continued to complain of numbness prompting electrodiagnostic testing 16 weeks after injury. Electrodiagnostic studies of the left median nerve at the wrist revealed absent sensory response and abductor pollicis brevis (APB) motor distal latency of 8.31 ms and amplitude of 1.9 mV consistent with severe median nerve neuropathy. Conservative management with nighttime splinting for eight weeks was attempted with no improvement in symptoms. 

On physical exam, the patient had a positive Tinel sign at the wrist. The Phalen’s test was negative. Static two-point discrimination was 10 mm at the volar thumb tip and <5 mm in all other digits. The patient reported normal sensibility at thenar eminence indicating that the palmar cutaneous branch was unaffected.

**Figure 3:**
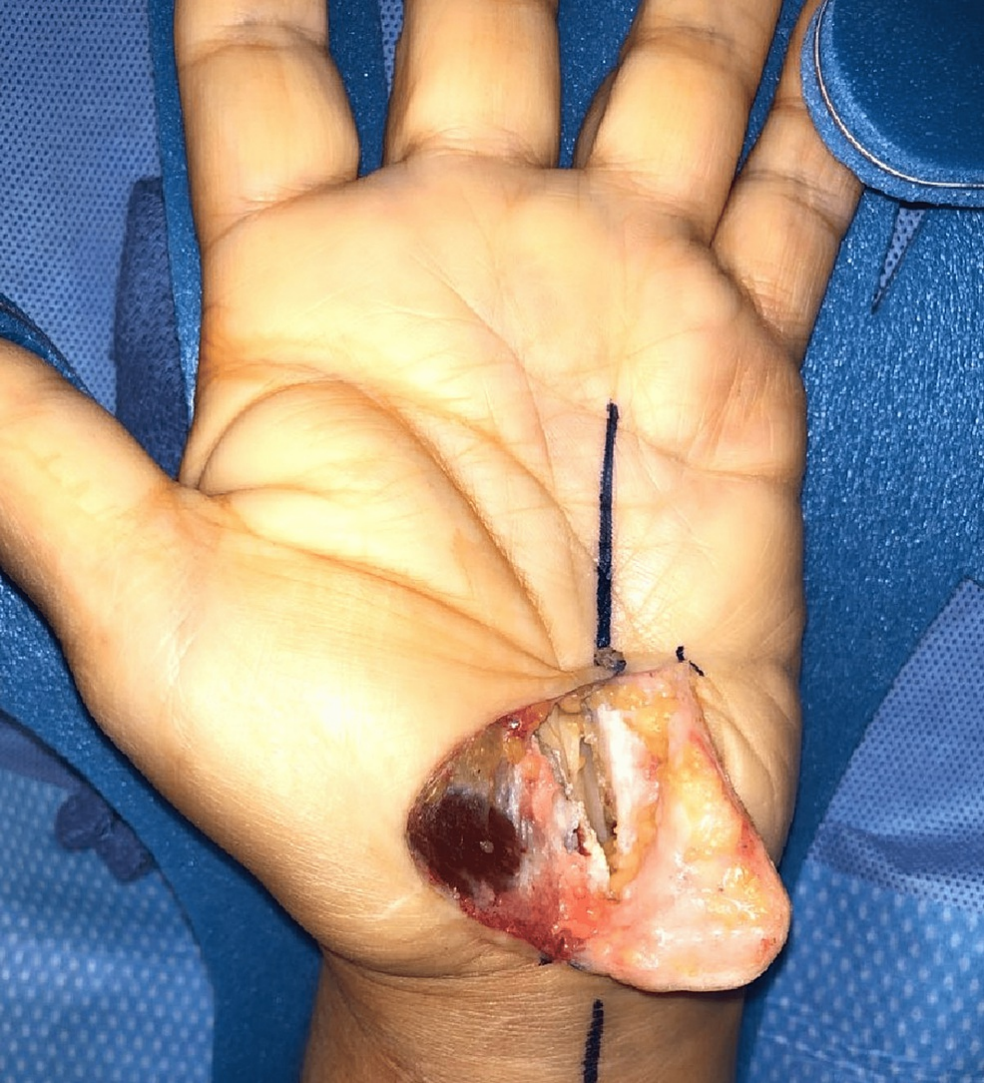
Visualization of the carpal tunnel Palmar flap opening that allowed the visualization of the carpal tunnel and median nerve.

**Figure 4:**
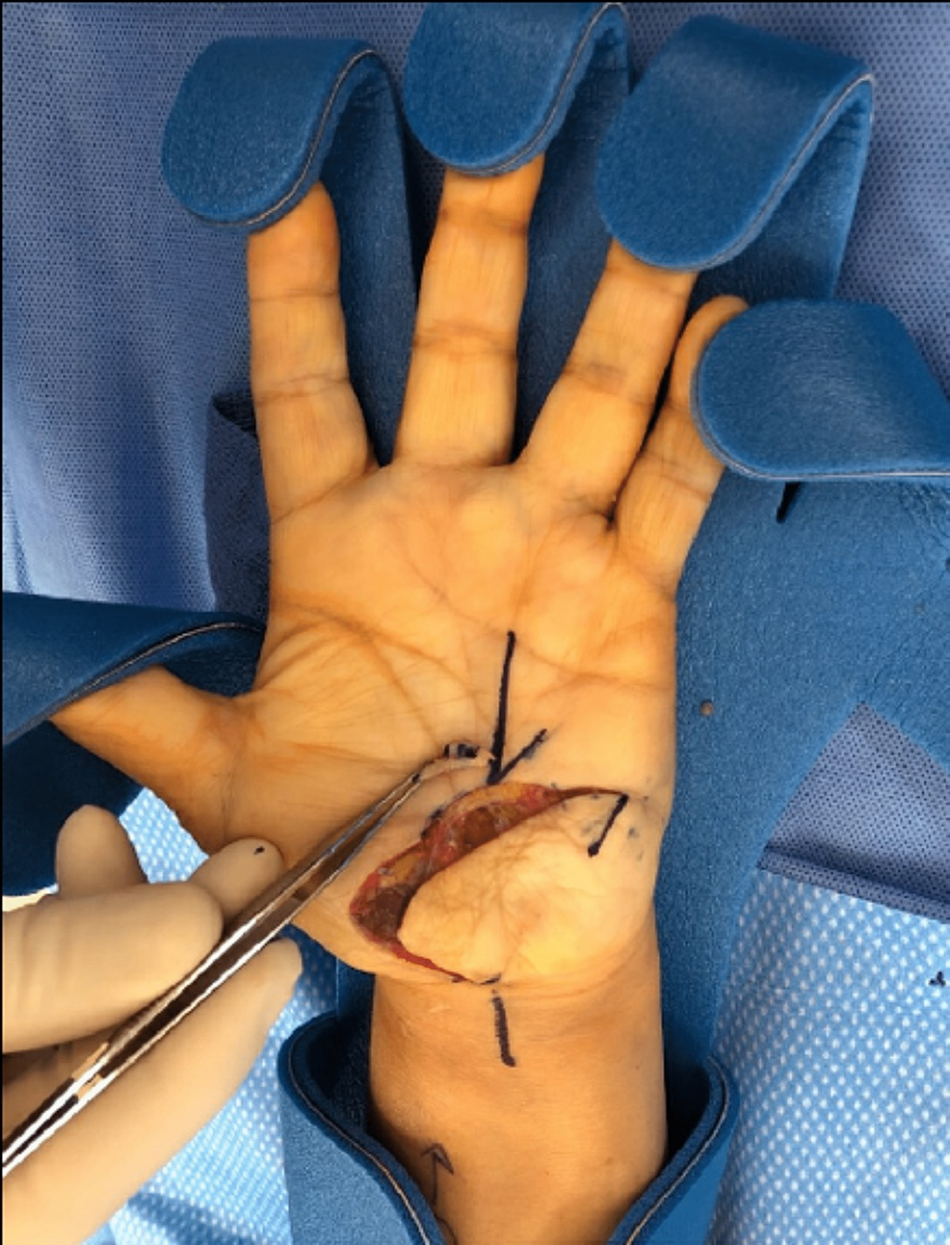
Surgical incision and markings Seen are the palmar incision along the previous scar. The scar was excised from the central palm with forceps. Also seen are the planned Z-plasty markings.

**Figure 5:**
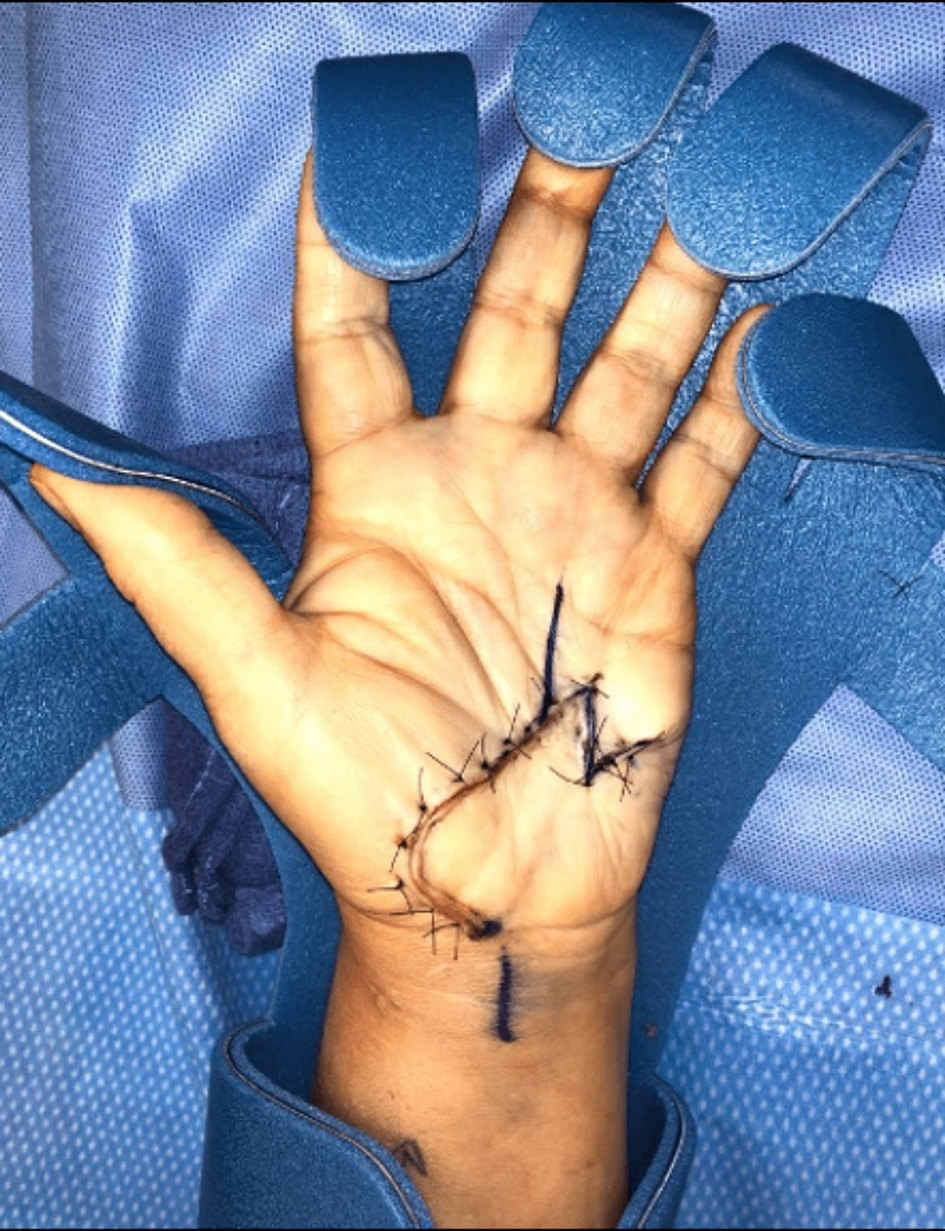
Closure after carpal tunnel release and transposition of Z-plasty limbs

The patient underwent surgical intervention for the management of scar contracture and CTS under general anesthesia. Given the scar and previous trauma to the palm, access to the carpal tunnel was not the standard approach. An incision was made along the prior scar, and the skin flap was elevated to provide wide visualization of the carpal tunnel (Figure 3). Using a combination of blunt and sharp dissection, the transverse carpal ligament was incised. Once decompressed, the median nerve was noted to have an hourglass configuration towards the distal half of the carpal canal contiguous with the thick band of scar tissue on the palm. Primary closure of the raised flap skin was not possible without recreating a tight band transversely across the palm. We designed a Z-plasty on the ulnar side of the scar, where the transverse tightness was most pronounced. After the hypertrophic tissue was excised, the surrounding skin was widely undermined (Figure 4). The Z-plasty had 60-degree limbs approximately 12 mm in size in the area of scar contracture. Once the limbs were elevated and undermined, we then transposed the Z-plasty limbs and sutured these down using interrupted 4-0 nylon sutures (Figure 5). Lastly, a small back cut was made at the base of the most ulnar Z-plasty limb to permit further advancement and rotation radially. 

**Figure 6:**
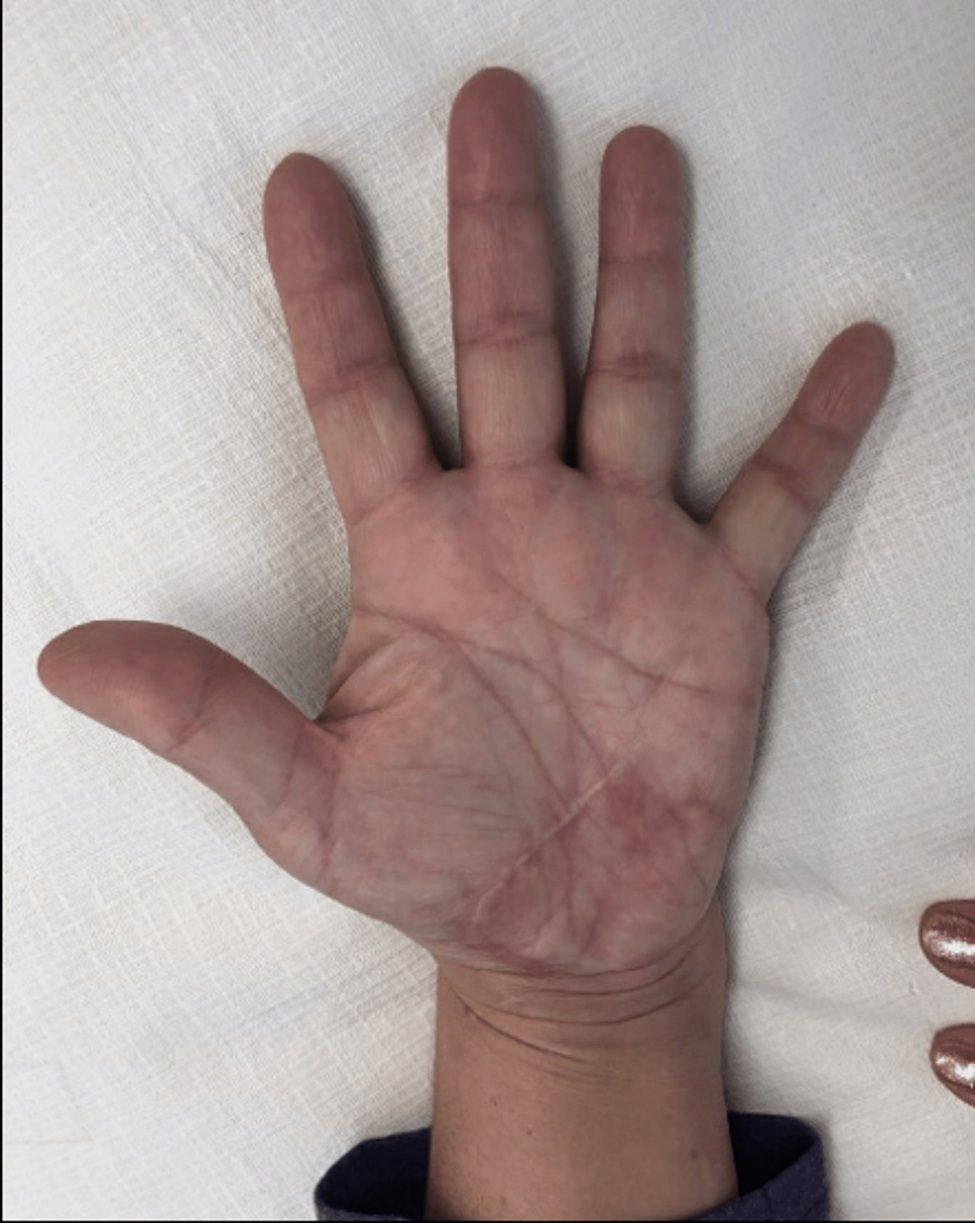
Three months postoperative image The patient no longer had limitations in thumb abduction and extension. The carpal tunnel symptoms had resolved.

The patient returned for suture removal two weeks after surgery. At the three-month follow-up appointment, there was a significant improvement in the active range of motion of her left thumb, resolution of median neuropathy symptoms, and there was no pain along the scar (Figure 6). Therefore, hand therapy was discontinued. 

## Discussion

Z-Plasty 

**Figure 7:**
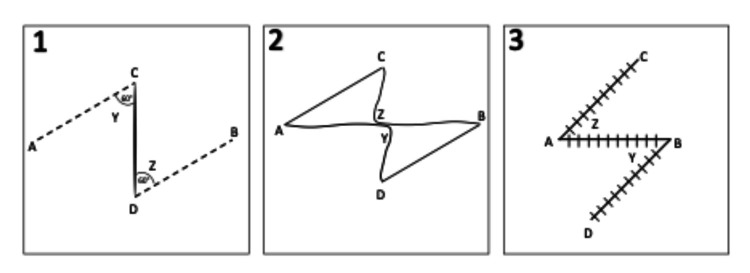
Z-plasty design The central limb (CD) is located along the scar. Lateral limbs AC and DB are drawn at 60-degree angles to scar at Y and Z.  Marks are incised and triangular flaps are elevated. The tip of flap Y is transposed to B and the tip of flap Z is transposed to A.

The Z-plasty is a versatile random pattern local transposition flap that relieves tension along scars. On the hand, a Z-plasty or permutations therein such as the 4-flap Z-plasty and jumping man flap classically have been deployed for web space and digital flexion contractures. Fundamentally, a Z-plasty lengthens and reorients scars to reduce tension (Figure 7) [2,3]. Other angles from 20 to 90 degrees can be used; however, deviating from 60 degrees comes with caveats. Angles less than 60 degrees are less difficult to transpose but provide less lengthening and reorientation. Angles larger than 60 degrees result in greater lengthening and reorientation but are more difficult to transpose [4,5]. Mathematically, theoretical gain in scar length can be calculated (Table 1). However, work by Furnas (&) Fischer in 1971 displayed that actual length gains were less in vivo than calculated [6]. They demonstrated that the actual gain of length was usually 55% to 84% of the theoretical gain of length. Inherent and differing skin tension in various anatomical regions is theorized to account for this difference [3,6].

**Table 1:** Z-plasty angles and theoretical gain in length as described by Furnas and Fischer

Z-plasty limb angle	Theoretical gain of central limb length
30°	25%
45°	50%
60°	75%
75°	100%
90°	120%

Median neuropathy from scar contracture 

The emergence of CTS symptoms four weeks after injury and a painful scar in our patient led us to believe that the fibrous adhesions instigated a traction-type extraneural neuropathy as described by Tos et al. [7]. Extraneural scarring impairs nerve gliding planes at rest or during elongation, which can result in reduced blood flow that induces nerve damage. In 1991, Hunter described traction neuropathy arising from a prior subacute trauma which resulted in the formation of fibrous attachments along the nerve that chronically impairs nerve gliding. Chronic traction-type injuries appeared to coexist with compression neuropathies, which were ultimately treated with decompression, not unlike the more widely known double crush phenomena [8,9]. Hunter’s observations were supported by later studies on rat sciatic nerve models for nerve repairs under tension by Clarke et al., where an 8% increase in nerve tension after repair induced a 50% reduction in nerve blood flow [10].

After an incomplete release of the transverse carpal ligament, the most common reason for recalcitrant CTS after surgical decompression is fibrous adhesions to the median nerve [11]. Multiple strategies have been developed to address extramural scarring around the median nerve including vein wrapping, synthetic collagen wrapping, fat grafting, and vascularized fat, synovium, or muscle flaps [11]. Ultimately, we did not employ any of these techniques since our patient had a de novo CTS. 

Although our patient’s scar contracture was from a laceration, burns are a well-established cause of acute and chronic CTS in patients with thermal hand burns. Burns produce extreme edema in the acute phase and hypertrophic scarring later owing to profound inflammatory and metabolic responses to thermal injury [12]. Acute edema from hand burns can cause acute CTS necessitating escharotomy. Median nerve compression may also occur in a delayed fashion while burns contract as they heal. In a review of burns patients presenting with compression neuropathies, the days from the date of injury to the date of release ranged from 46 to 614 (average 264 days) [13]. Our patient, who did not suffer a thermal injury, had carpal tunnel symptoms emerge one month after injury and underwent release 295 days after injury.

There are additional risk factors for CTS. Traumatic causes include upper extremity injury such as perilunate dislocation and distal radius fractures. Certain medical conditions such as pregnancy, diabetes, and hypothyroidism are also linked to CTS. These conditions contribute to increased pressure in the carpal tunnel [14].

## Conclusions

Our case provides a unique presentation and management of a palmar laceration causing limited thumb range of motion and CTS. The Z-plasty provided tension relief along the scar and restored normal thumb movement, and the decompression of the median nerve resolved the patient’s neuropathy symptoms. 
